# *Plasmodium falciparum* genotype and gametocyte prevalence in children with uncomplicated malaria in coastal Ghana

**DOI:** 10.1186/s12936-016-1640-8

**Published:** 2016-12-09

**Authors:** Ruth Ayanful-Torgby, Akua Oppong, Joana Abankwa, Festus Acquah, Kimberly C. Williamson, Linda Eva Amoah

**Affiliations:** 1 Noguchi Memorial Institute for Medical Research, University of Ghana, Accra, Ghana; 2 Uniform Services University of the Health Sciences, Bethesda, Maryland USA

**Keywords:** Gametocytes, Genetic diversity, Multiplicity of infection

## Abstract

**Background:**

*Plasmodium falciparum* gametocytes are vital to sustaining malaria transmission. Parasite densities, multiplicity of infection as well as asexual genotype are features that have been found to influence gametocyte production. Measurements of the prevalence of *Plasmodium* sp. gametocytes may serve as a tool to monitor the success of malaria eradication efforts.

**Methods:**

Whole blood was collected from 112 children aged between 6 months and 13 years with uncomplicated *P. falciparum* malaria attending three health facilities in southern Ghana from June to August, 2014 before (day 0) and 4 days after completion of anti-malaria drug treatment (day 7). Malaria parasites were observed by microscopy and polymerase chain reaction (PCR); submicroscopic gametocyte carriage was measured by a *Pfs25* (PF3D7_1031000) mRNA real time reverse transcriptase polymerase chain reaction (RT-PCR). Parasite genotyping was performed on gDNA extracted from dried filter paper blood blots by amplification of the polymorphic regions of *msp1* (PF3D7_0930300) and *msp2* (PF3D7_0206800) using PCR.

**Results:**

Microscopy estimated 3.1% (3/96) of the total population to carry gametocytes on day 0, which decreased to 2.1% (2/96) on day 7. In contrast, reverse transcriptase-real time PCR (RT-PCR) analysis of a subset of 35 samples estimated submicroscopic gametocyte carriage to be as high as 77% (27/35) using primers specific for *Pfs25* (CT < 35) on day 0 and by day 7 this only declined to 60% (21/35). Genotyping the *msp2* gene identified higher levels of MOI than the *msp1* gene.

**Conclusions:**

Although below detection by microscopy, gametocyte prevalence at submicroscopic levels are high in this region and emphasize the need for more effective elimination approaches like the development of transmission-blocking vaccines and safer gametocytocidal drugs.

**Electronic supplementary material:**

The online version of this article (doi:10.1186/s12936-016-1640-8) contains supplementary material, which is available to authorized users.

## Background

In Ghana, malaria is still one of the leading causes of outpatient attendance and mortality in children under the age of 5 years [1], despite enhanced control efforts. *Plasmodium falciparum*, the most lethal of the five species that cause human malaria, is responsible for about 90% of all malaria cases in Ghana [2]. Malaria transmission requires the production of sexual stage parasites that are stimulated to fertilize after being taken up during a blood meal by a mosquito [3]. The zygote continues development in the mosquito producing an oocyst containing sporozoites that can initiate an infection in humans during a subsequent blood meal. Sexual reproduction coupled with high genetic diversity in the local parasite population and concurrent infections with polymorphic parasite lines provides genetic flexibility that allow adaptation to immune and drug pressure [4] and also influences malaria transmission success [5]. For example, an increase in the rate of sexual recombination has been found to give rise to parasites with different drug resistant profiles [6–9]. Low haematocrit and history of prolonged illness have been associated with gametocyte prevalence detected using microscopy [10]. Genetic factors are also likely to play a role since gametocyte production and mosquito infectivity have been shown to vary between parasite lines [11–14]. Together the dynamics of parasite diversity and gametocyte production have important implications for the acquisition of immunity by the host and the spread of drug resistant parasites. However, monitoring gametocyte production in the human host is complicated by low production levels and sequestration of immature gametocytes during the 10–12 days required for the development of stage V *P. falciparum* gametocytes. Only mature stage V gametocytes circulate and can be detected in peripheral blood. Previous work in East Africa and Asia demonstrated that gametocytes are resistant to artemisinin-based combination therapy (ACT) and, consequently, patients remain infectious for over a week after asexual parasite clearance [15, 16]. The role of the immune response in controlling gametocyte levels in the human host has not been well established [17]. However, *Pfs230* and *Pfs48/45* are expressed on the gametocyte surface during development in the RBC in the human host [18–20] and anti-Pfs230 and Pfs48/45 antibodies are generated during a natural infection [19–24] and thus can serve as a marker for recent gametocyte exposure.

This study assessed the prevalence of submicroscopic gametocytes levels and asexual parasite diversity in patients aged between 6 months and 13 years with uncomplicated *P. falciparum* infections. Understanding these patterns is critical to the development of intervention strategies in high transmission areas. The persistence of gametocytes in children with uncomplicated malaria 4 days after a 3-day anti-malarial drug course (day 7) was also analysed.

## Methods

### Ethical considerations

The study was approved by the Institutional Review Board of the Noguchi Memorial Institute for Medical Research (NMIMR) and Ghana Health Services. Before recruitment each parent/guardian was informed of the objectives, methods, anticipated benefits and potential hazards of the study. The parents/guardians were encouraged to ask questions about any aspect of the study that was unclear to them and informed about their liberty to withdraw their children at any time without penalty. Children were enrolled only after written parental consent had been obtained. All patient information is treated as confidential.

### Study site and population

**Fig. 1:**
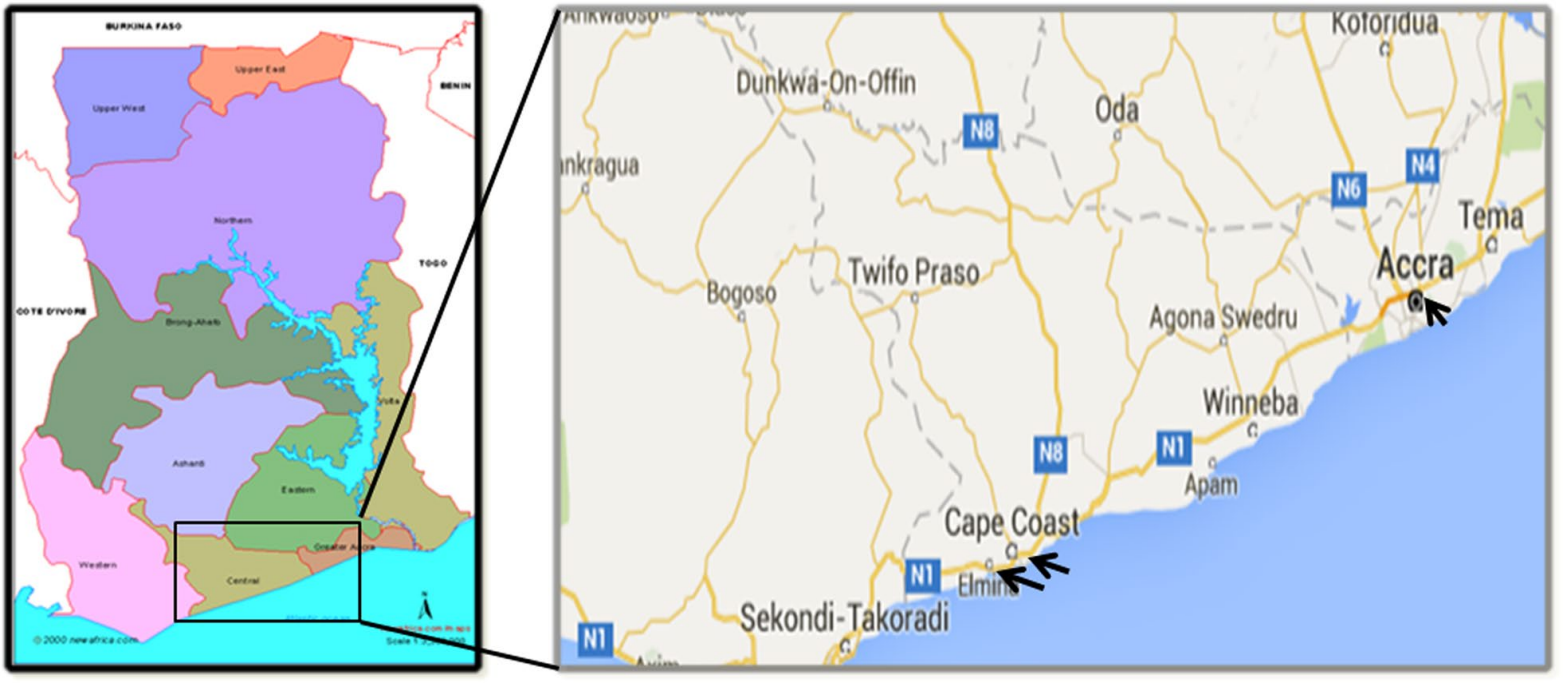
A geographic map showing location of the study sites in Southern Ghana

The study was conducted in three health facilities, the Ghana Atomic Energy Commission (GAEC) Clinic in Accra, Ewim Health Centre and Elmina Health Centre, both in Cape Coast. Cape Coast (05°05′ N, 01°15′ W), an urban setting, has an estimated population of 227,269 and lies in the Coastal savannah region (Fig. 1). Cape Coast, which is the capital of the Central Region, is about 165 km from Accra. Malaria transmission in this area is perennial with most of the disease occurring during the major rainy season in June/July. Accra (05°35′ N, 00°06′ W), an urban setting has an estimated population of 2,291,352. Accra is the capital city of Ghana with a total land area of 201 sq km. Accra is one of the most populated and fast growing metropolis in Africa with an annual growth rate of 4.3% [25] and lies in the coastal savannah region. Malaria transmission in this area is also perennial with most of the disease occurring during the major rainy season in June/July. The study group comprised of 112 children between the ages of 4 months–13 years, where 55 participants where between 8 and 60 months and the rest between 72 and 156 months (Table 1). The malaria patients enrolled in this study were prescribed ACT (artemether-lumefantrine) at the health centre, BUT there was no evaluation/monitoring of ACT intake.

**Table 1:** Background data of study participants

Parameter	Values
Age range (months)	4–156
Geometric mean age (months)	52.18
D0 parasite prevalence by microscopy^a^	72/96
D0 parasitaemia range (per µl of blood)^a^	400–648,080
Geometric mean D0 parasitaemia (per µl of blood)^a^	20,319
D7 parasite prevalence by microscopy^a^	1/96
D0 gametocyte prevalence by microscopy	3/96
D7 gametocyte prevalence by microscopy	2/96
D0 parasite prevalence by PCR	96/96
D7 parasite prevalence by PCR	3/96
G6DP	
Normal	100/105
Deficient^b^	5/105

^a^Asexual parasite prevalence

^b^G6PD Deficient: A376G plus one or more (G202A, G680T or T968C) mutation. Only 105 out of 112 could be genotyped

### Sampling

A total of 112 samples were collected from patients aged 6 months–13 years, who visited the health centre with uncomplicated malaria from June to August 2014. Children who were found to be *P. falciparum* positive by microscopy were enrolled after parental consent was obtained and in accordance with the study inclusion criteria [26]. Sixteen of the children did not return for the 1 week follow up visit. Prior to treatment (day 0) and during the 7 day follow up visit (day 7), 2 ml of venous blood was collected into EDTA vacutainer tubes and an aliquot spotted onto filter paper (Whatman^®^ 3 mm). The filter paper was air-dried and stored desiccated at room temperature. The EDTA samples were immediately centrifuged and the plasma collected and saved for future use. One hundred microlitres of pelleted cells were preserved in 500 µl of Trizol (Tri Reagent, Invitrogen). All the samples were transported to the NMIMR for analysis.

### Parasite density

Thin and thick blood smears were prepared from capillary blood collected on day 0 and venous blood collected on day 7. The thin smears were used for *Plasmodium* species identification and thick smears used for parasite (asexual and sexual) density estimation by using 100X oil immersion light microscopy. *Plasmodium falciparum* parasites were counted per 200 leukocytes to estimate the parasite density per microlitre of blood. All blood smears were read by two independent microscopists.

### Extraction, purification and analysis of parasite RNA

Thirty-five day 0 and day 7 paired trizol preserved samples were selected based on their antibody titres against a gametocyte specific antigen Pfs48/45-6C (19). The Pfs48/45 titres were obtained during an experiment that will be published separately and the selected samples included sero negative, low seropositive and high sero positive relative to a positive control. RNA was isolated from samples (100 μl packed blood cells) using the Quick RNA MiniPrep kit (Zymo Research) following the manufacturer’s protocol, which included an on column DNaseI treatment prior to elution with 36 μl of elution buffer. Eight microlitres of RNA extracted from the day 0 and day 7 blood samples were converted into 20 μl of cDNA using a Qiagen Omni script Reverse Transcriptase kit (Qiagen) and oligo-dT primers. To check for genomic DNA contamination after RNA extraction, conventional PCR was performed on all RNA (4 µl) samples and 2 µl of their corresponding cDNA samples diluted 1:10. Controls used in these PCR reactions were *P. falciparum* and *Homo sapiens* genomic DNA as well as a no template control (NTC) from the cDNA conversion reaction. Pfs48/45 primers (Additional file 1) were used for the day 0 samples and human blood group O genotyping primers as described by Tun et al. [27] (Additional file 1) were used for the day 7 samples that, except for one patient, were no longer positive for *P. falciparum* asexual parasites by microscopy. Real time (RT)-PCR was only carried out on cDNA samples after confirming that the PCR products run on 2% ethidium bromide stained agarose gels did not have amplified products in wells with the NTC and the RNA samples but there were products in the cDNA and 3D7 gDNA samples. A two-step SYBR Green 1 non-quantitative real time reverse transcription-PCR (RT-PCR) was performed on the *P. falciparum* cDNA isolated from the day 0 and day 7 blood samples.

Gametocyte carriage was assessed using *Pfs25* transcript levels. Validation of the *Pfs25* mRNA primer set (Additional file 1) was performed on cDNA converted RNA extracted from cultured NF54 stage IV and V gametocytes using the same methods describe above for the patient samples. The cDNA was diluted 1:20 with subsequent twofold serial dilution until 1: 640 and each dilution was tested in triplicate using a Pfs25 primer concentration of 300 nM and the fast SYBR^®^ Green 2X master mix RT-PCR kit (Applied BioSystems). The reaction was run on an Applied Biosystems One-step Plus RT-PCR machine and the cycling conditions were 95 °C for 20 s, 40 cycles of 95 °C for 3 s and 60 °C for 30 s. A melt curve was performed on the final product. Applied Biosystems StepOnePlus software was used to determine the threshold cycle (CT) for each cDNA concentration and the data used to plot a standard curve (Additional file 2). The CT values of the no template control was used to determine the cut-off for the presence or absence of gametocytes. The patient samples were tested in triplicate using Fast SYBR^®^ Green 2X master mix RT-PCR kit (Applied BioSystems). Two microlitres of cDNA diluted 20 fold (equivalent to 0.11 µl of packed blood cells) in a total reaction volume of 20 µl. The same fast SYBR^®^ Green RT-PCR conditions described above were used and the data analysed using Applied Biosystems StepOnePlus software.

### Extraction of parasite DNA

Genomic DNA was extracted from two 3 mm punches of dried blood blots using Saponin-Chelex extraction [28]. Briefly, the blood stained filter paper discs for each sample (day 0 and day 7) were incubated in 1.5 ml containing 1120 μl of 0.5% saponin/PBS solution overnight at room temperature on a shaking incubator. After the overnight incubation, the supernatant was discarded and the samples washed twice with 1 ml PBS, followed by a high-speed centrifugation at 10,000×*g*. Finally, 150 μl of a 6% Chelex (Sigma-Aldrich, USA) in DNase/RNase free water was added to the washed samples and incubated at 95 °C for 5 min to extract DNA from the samples. After a final high-speed centrifugation, the supernatant containing the DNA was stored at −20 °C until used for the genotyping amplification reactions.

### Molecular identification and genotyping

To distinguish three major allelic families (K1, MAD 20, and RO33) block 2 of *msp1* and the two allelic families (FC27 and IC3D7) central polymorphic region of *msp2*, nested PCR was performed using family specific primers [29] shown in Additional file 1. All amplification reactions were carried out in a final volume of 15 μl. The outer PCR reaction mix contained 200 nM dNTP, 2 mM MgCl_2_. 133 nM of each primer, and 0.5 unit of One Taq DNA polymerase (New England BioLab) in addition to 4 μl (about 0.25 μl of whole blood) of genomic DNA (gDNA) template. In the nested reaction, 0.5 μl of the outer PCR product was used as template in a PCR reaction mixture containing 200 nM dNTP, 1.8 mM MgCl_2_. 200 nM of each primer and 0.5 unit of One Taq DNA polymerase. Each amplification profile consisted of initial denaturation at 94 °C for 3 min, followed by 30 cycles at 94 °C for 1 min; 50–59 °C (depending on the primer pair annealing temperatures) for 35 s, and 68 °C for 2.5 min; with final extension at 68 °C for 3 min. The PCR reaction mixtures were run on a thermal cycler (MJ Research Tetrad PTC-225 Thermal Cycler, USA). Allelic specific positive controls 3D7, K1, HB3 and RO33 gDNA and no template negative controls were included in each set of reactions. PCR products were separated using 2% ethidium bromide-stained agarose gels respectively and visualized under UV illumination.

### Multiplicity of infection

The multiplicity of infection (MOI) or number of genotypes per infection was calculated by dividing the total number of *msp1* or *msp2* fragments detected by the number of samples positive for the same marker. Samples with more than one genotype were considered as containing multiclonal infections while the presence of a single allele was considered as clonal infection.

### G6PD genotyping

PCR based G6PD genotyping was performed on the extracted DNA using primers listed in Carter, et al. as previously reported [30]). The A376G mutation was characterized in each DNA sample using restriction fragment length polymorphism (RFLP) by digesting the 376 PCR amplicon with 1 unit of FOKI restriction enzyme at 37 °C for an hour. Only samples with the 376G genotype where further analyzed for three other sub-Saharan African cDNA mutations, G202A, G680T and T968C also using RFLP as described in our previous work [31]. The G202A PCR amplicon was digested with NlaIII restriction enzyme, the G680T amplicon was digested with BstNI restriction enzyme and the T968C amplicon digested with NciI restriction enzyme for 1 h at 37 °C. All the PCR fragments and the digested fragments were viewed under UV light after resolving on a 2% agarose gel containing 0.5 µg/ml ethidium bromide.

### Data analysis

Data were entered and analysed using Excel and GraphPad Prism version 7.0. The Shapiro–Wilk normality used to determine if the data was normally distributed. The data was not normally distributed, thus the Mann–Whitney test was used to determine relationships between age and PD, MOI and Pfs25 RT-PCR CT values using GraphPad Prism v7.0. The geometric means and other column statistics were obtained using GraphPad Prism v7.0. Proportion was used to present the distribution of different allelic families. The frequency of *msp1* and *msp2* family alleles was calculated as the ratio of the number of PCR products obtained for each family to the total number of gene specific PCR products identified. A sample was classified as harboring a multiclonal infection when more than one amplified fragment was obtained during either or both the *msp1* and *msp2* genotyping. The mean MOI was calculated as a total number of *P. falciparum* genotypes detected per total number of positive samples. Linear regression was used to determine the relationship between MOI and day 0 and day 7 CT values obtained during the Pfs25 real time reverse transcriptase PCR. Statistical significance was defined as *P* ≤ 0.05.

## Results

Asexual and gametocyte parasite density in the study participants were monitored by microscopy on day 0 and day 7, 4 days after a 3 day ACT regimen. All the children were PCR positive for *P. falciparum* after MSP genotyping, however after the thick smears prepared by the hospital laboratory staff were re-read by highly trained microscopists, only 72 of the children had microscopy confirmed parasites on day 0. The geometric mean of *P. falciparum* parasite density recorded by microscopy for the 96 participants that returned for the follow up visit was 20.319/µl blood (95% CI 13.568–30.428) at day 0. Three samples were positive for *P. falciparum* by MSP genotyping on day 7, although only one sample had microscopy confirmed asexual parasites with a density 76,080/µl.

Three samples were positive for gametocytes by microscopy on day 0 with a mean gametocyte density of 93.33 (SEM 35.28) two of these samples remained gametocytaemic on day 7, with gametocyte densities of 320 and 400/µl. *Plasmodium falciparum* parasite carriage measured by PCR analysis of the MSP2 and MSP1 genes revealed that all the day 0 samples were positive for *P. falciparum* parasites but only three day 7 samples were positive. MSP genotyping revealed one day 7 sample to be a recrudescent infection and the other two as new infections. The geometric mean participant age was 52.18 (95% CI 44.74–60.86) months, with a range from 4 months through to 156 months. No significant relationship between age and PD was identified in this study (Additional file 3). The prevalence of G6DP deficiency was estimated at 4.76% (5/105) and consisting of three hemizygous A- males and two AA- heterozygous females in the entire study population, however no data was available for seven of the children (Table 1).

### Submicroscopic gametocytes

The *Pfs25* RT-PCR was used to screen for submicroscopic levels of gametocytes in 35 trizol preserved samples that were selected to represent a range of titres for gametocyte specific antigen Pfs48/45-6C (Additional file 4). Although *Pfs25* mRNA is not translated until uptake by a mosquito, the transcript is expressed and stored in mature female gametocytes [32] and it has been developed as a sensitive and specific marker for circulating mature gametocytes [33, 34]. Twenty-seven of the day 0 samples were classified as positive using a CT cut off of 35 (77%) (Table 2; Additional file 4). Eighteen of the 35 samples were gametocyte positive on both days (51.4%), including the sample that was gametocyte positive by microscopy on day 0 and day 7. Three samples were identified that were gametocyte negative on day 0 but gametocyte positive on day 7, making the total number of gametocyte positive samples for day 7 equal 21 (60%). Four samples which were gametocyte negative on day 0 and remained gametocyte negative on day 7 (Table 2; Additional file 4). Interestingly, significantly more children above the age of 5 years had submicroscopic gametocytes on day 7 (Additional file 3).

**Table 2:** RT-PCR detection of submicroscopic gametocytes

*Pfs25* cDNA	Day 0	Day 7
Positive	27	21
Negative	8	14
Total	35	35

All cDNA samples were positive for a human blood group gene by RT-PCR. D0 Samples negative for *Pfs25* were RT-PCR positive for KAHRP

### Genetic diversity and multiplicity of infection

**Fig. 2:**
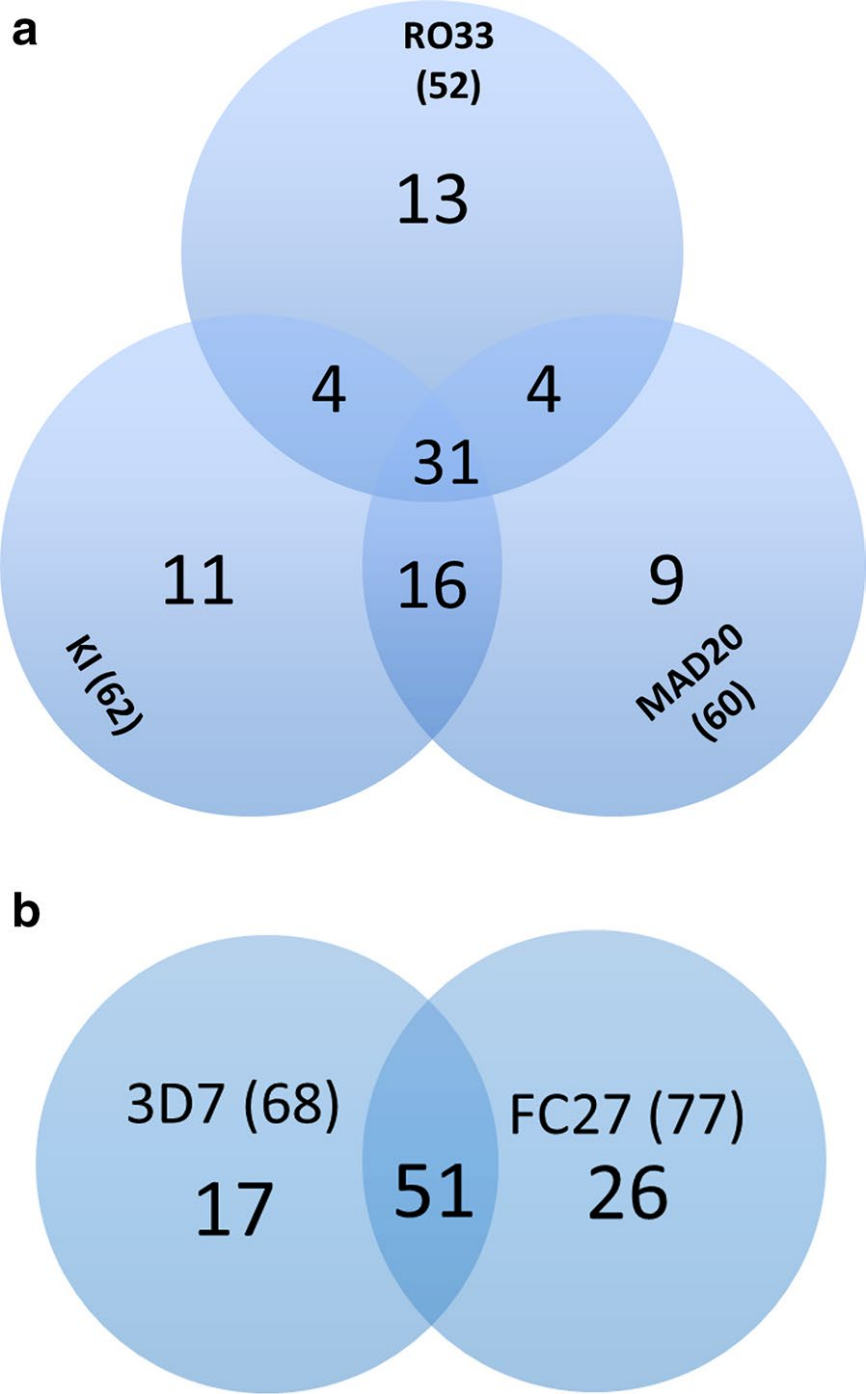
Representation of *msp1* (**a**) and *msp2* (**b**) allele families in the study population. The distribution of parasites within the major families and their combinations in patient samples are shown. The *numbers* in *brackets* represents the total number of samples that contained at least one parasite belonging to the allelic family

**Fig. 3:**
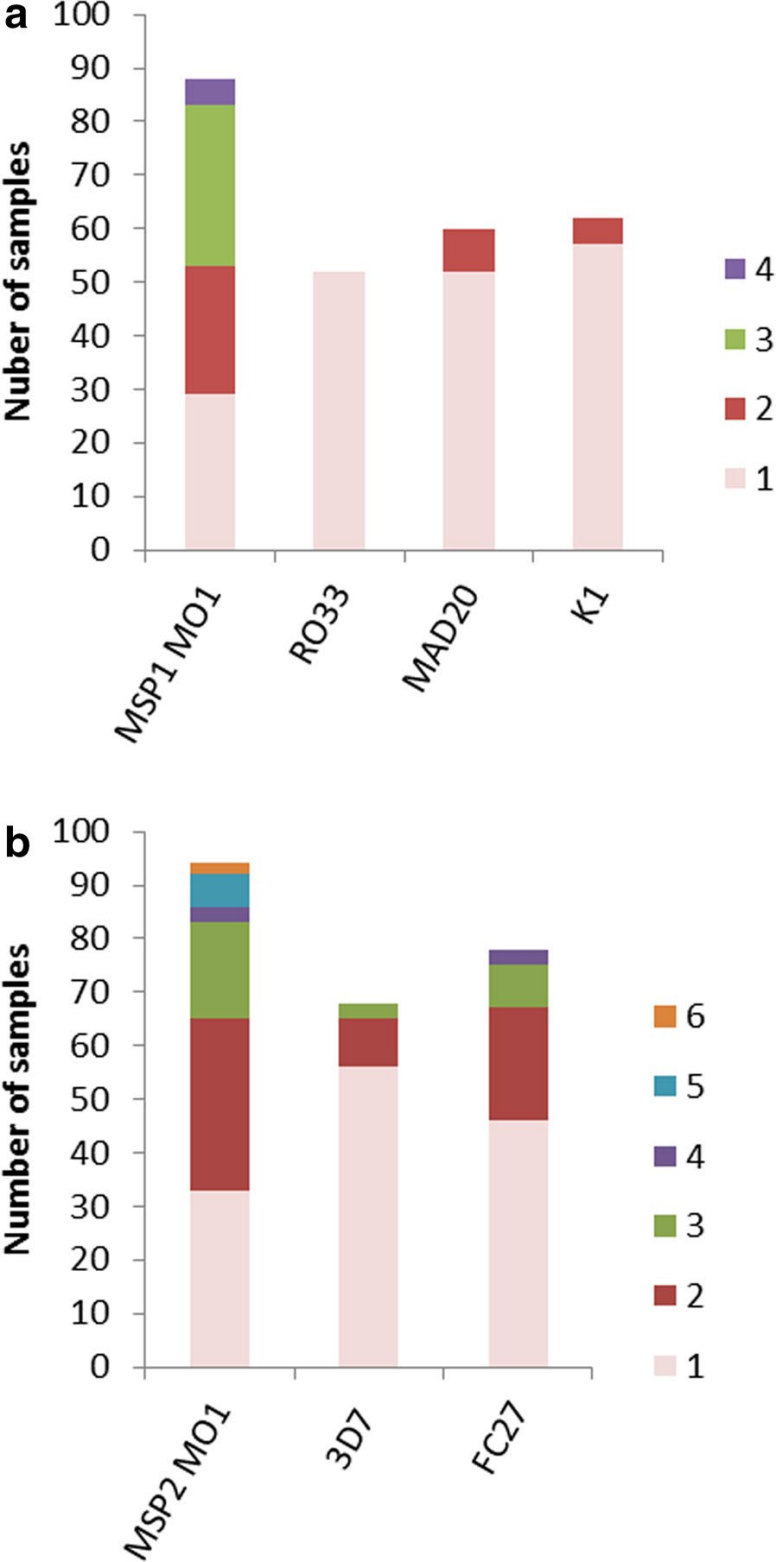
Prevalence and multiplicity of infection of *msp1* (**a**) and *msp2* (**b**) alleles. Each distinct amplicon produced by *msp1* or *msp2* family specific PCR represents a particular parasite clone. The number of samples that contained distinct alleles (color coded and labelled 1 through 6) for **a** *msp1* (*msp*1 MOI) or each of the three *msp1* families (RO33, MAD20 or K1) or **b** *msp2* (*msp*2 MOI) or each of the two *msp* families (3D7 or FC27) are plotted

*Plasmodium falciparum* genes, which show extensive polymorphisms, such as merozoite surface proteins 1 (*msp*1) and 2 (*msp*2) can be used as markers to study parasite genetic diversity and multiplicity of infection (MOI) [35]. *Msp1* Block 2 is the most polymorphic region of the gene and is grouped into three allelic families namely K1, MAD 20, and RO33 type, while in the *msp2* gene block 3 is the most polymorphic region and consists of FC27 and 3D7 families [36]. In total, PCR products were detected using at least one *msp1* and *msp2* family specific primer set in 86 of the 96 samples (Table 3). Only 12 samples had a single band for both *msp1* and *msp2*, indicating that less than 14% of the subjects had monoclonal infections (as predicted by *msp* genotyping). The remaining samples had a range of combinations of the different allelic families. For *msp1*, the frequency of MAD20 and K1 family alleles were similar and higher than the R033 alleles (Fig. 2a). Of the 88 samples with PCR products for *msp1*, 62 had at least one K1 allele, 60 had at least one MAD20 allele and 52 had at least one R033 allele. Thirty-five percent of the subjects were multiply infected with parasites from all three *msp1* families. Additional diversity was also evident within the K1 and MAD20 families, as eight subjects had two distinct MAD20 alleles and five samples had two distinct K1 alleles (Fig. 3a). Five samples had 4 distinct *msp1* alleles, 30 had 3 alleles, and 24 had 2 alleles, while 29 were monoclonal for *msp1* (Fig. 3a). Fifty-nine percent of the *msp1* monoclonal infections belonged to the R033 family (13 of the 29). The geometric mean MOI for *msp1* in the study was 1.90 (95% CI 1.71–2.11).

**Table 3:** Prevalence of clonal parasite infections in the samples

Parameter	RO33	MAD20	K1	3D7	FC27	Clonal^a^
*msp1* (88)	13	7	9			29
*msp2* (94)				15	18	33
*msp1* + *msp2* (86)	4	5	3	7	5	12

^a^Parasite population within a sample as determined by MSP1 and MSP2 family specific PCR. Two samples which failed *msp2* genotyping PCR yielded products in *msp1* genotyping and as such there were 86 samples common to both genotyping procedures. The numbers in brackets represents the total number of samples in group

For *msp2*, 77 of the 94 samples with *msp2* PCR products had at least one FC27 allele and 68 had at least one 3D7 allele. Again most samples were polyclonal, containing alleles from both families (51 of 94 samples) (Fig. 3b). As with *msp2*, some samples also contained multiple alleles within one *msp2* family (Fig. 3b); with one sample containing six distinct *msp2* alleles. Only thirty-three of the 94 PCR-positive *msp2* samples were monoclonal for *msp2*. The geometric mean of *msp2* MOI was 1.88 (95% CI 1.68–2.10).

Combining genotyping results, the study identified only 12 samples that carried a parasite population with a single allele for both *msp1* and *msp2* (Table 3), suggesting that the clonal parasite population estimated solely by *msp1* genotyping is overestimated by 58.6% (17/29) and 63.6% (21/33) when *msp2* genotyping is used exclusively. Two samples that were multiclonal by *msp1* genotyping did not have any *msp2* data and eight samples, three clonal and five multiclonal identified by *msp2* genotyping did not have *msp1* data. No significant correlation between the MOI for either *msp1* or *msp2* and submicroscopic gametocyte prevalence was found in this study (Additional file 3).

## Discussion

The high prevalence (77%) of mature gametocytes detected by RT-PCR in the day 0 samples (Table 1) is consistent with the high malaria transmission rates observed in southern coastal Ghana [25] and is similar to studies in other regions of Africa using *Pfs25* RT-PCR [2, 16, 37–40]. The continued persistence of gametocytes in 60% of the patients even after the clearance of asexual parasites with ACT drug treatment, including 2 of the 3 patients with microscopically detectable gametocytes on day 7, highlights the lack of efficacy of ACT against mature sexual stage parasites [31, 38, 41, 42] and the need to develop new strategies to block the spread of malaria.

Although age was not significantly associated with submicroscopic gametocytes levels on day 0 (Additional file 3), older patients had a significantly higher prevalence of submicroscopic gametocytes on day 7 compared with the younger children due to them having a lower mean CT value for Pfs25 real time reverse transcriptase PCR value (Additional file 3), suggesting an age associated decrease in gametocyte clearance following ACT. A larger study that includes a wider age range of patients would be needed to confirm this and to begin to define the contributing factors, such as the role of a maturing immune response in gametocyte clearance. The acquisition of immunity to clinical malaria is well established [43], but immune mediated clearance of gametocytes has been more difficult to demonstrate [44].

Primaquine (PQ) is currently the only WHO approved drug with potent transmission-blocking activity that targets mature *P. falciparum* gametocytes [45–47]. As Ghana targets to move beyond the malaria control phase into the pre elimination phase, there is a possibility of implementing PQ with first-line anti-malarial ACT to reduce gametocyte prevalence in Ghana as is on-going in some countries in the elimination and pre elimination phase [45]. The prevalence and extent of g6pd deficiency is a major concern for malaria eradication programmes, where they plan to use PQ as a gametocidal agent as although a single low dose of PQ has been suggested to be well tolerated in G6PD deficient malaria patients, PQ has been found at certain concentrations and in certain instances to cause RBC lysis in g6pd deficient individuals [48–50]. The 4.7% incidence of g6pd deficiency identified in this study was similar to another study conducted in 2015 in two different communities along the coast of Ghana where G6PD deficiency was 5.9% in hemizygous males and homozygous females [31]. The prevalence of this population needs to be taken into consideration when evaluating the use of primaquine or other 8-aminoquinolines as gametocidal agents in this region.

Multiplicity of infection within the allelic families reduced clonality of the parasite population from 37.5 to 33% in *msp1* and 46 to 35% in *msp2* (Fig. 2; Table 3) and it possible that even higher MOIs would be identified within the allelic families if a more sensitive sizing technique such as capillary electrophoresis was used instead of agarose gel electrophoresis. High parasite MOI (≥2) has been associated with increased parasite density, although whether this is related to parasite survival or increased production was not directly evaluated [51]. Multiclonal infections also enhance the chance of heterozygous mating after the parasites have been taken up in a blood meal of a mosquito [4–7]. Fertilization between two distinct parasites provides the opportunity for chromosome recombination during meiosis, which enhances genetic diversity in the parasite population. Geometric mean MOIs for *msp1* and *msp2* of 1.90 and 1.88 were slightly lower than obtained in an earlier report from the middle belt of Ghana where MOI was 2.3 in July/August, [52] but similar to another report of MOI in southern Ghana which was 1.93 [24] and 1.3 in 2013 [53]. This discrepancy may be due to differences in ecological zones [54], malaria transmission intensity [36, 55] and/or the treatment-seeking behaviour of malaria patients within the various population [56, 57].

Even at these relatively low MOIs the majority of the samples (86%) contained more than one *msp1* or *msp2* allele (Fig. 3) indicating the potential for cross-fertilization once gametocytes are taken up by a mosquito. Gametocytes prevalence was not associated with MOI, indicating that multiply infected individuals were just as likely to be gametocyte carriers as those with monoclonal infections. The combination of high gametocyte prevalence and multiclonal infections sets the stage for mating between two distinct parasite lines and further enhancing parasite diversity.

## Limitations

Although all the volunteers were prescribed and administered received the same ACT, artemether-lumefantrine, they did so in their respective homes and as such compliance to the prescribed regimen could have been compromised. Such non-conformity in prescribed drug intake could influence the prevalence of both asexual and sexual stage parasites. This scenario reflects what happens in the community, where non-compliance can be a major obstacle for malaria control programmes.

## Conclusion

This study provides critical information on factors that influence malaria transmission such as the presence of submicroscopic levels of gametocytes in 77% of the children on day 0 which persisted in 60% of the children on day 7. The high prevalence of gametocytes and multiclonal infections (86%) in the children suggests there is ample opportunity for recombination during fertilization, which would enhance genetic diversity and could contribute to the emergence of drug resistant parasites. Older children were more likely to have submicroscopic gametocytes on day 7 suggesting an age associated decrease in gametocyte clearance following ACT treatment. The high prevalence of submicroscopic gametocytes levels is consistent with other studies and it will be important to monitor drug resistance, particularly ACT clearance in future studies.

## Additional files

**Additional file 1.** Primer list

**Additional file 2.** Validation of the Pfs25 mRNA primer set

**Additional file 3.** Statistical analysis

**Additional file 4.** Features of samples analyzed for submicroscopic gametocytes

## Abbreviations

ACT: artemisinin-based combination therapy
cDNA: complementary deoxyribonucleic acid
DNA: deoxyribonucleic acid
EDTA: ethylenediamine tetra acetic acid
GLURP: glutamate rich protein
MOI: multiplicity of infection
MSP: merozoite surface protein
RNA: ribonucleic acid
RT-PCR: real time reverse transcription polymerase chain reaction
