# Supplementary material for: Plasmodium falciparum genotype and gametocyte prevalence in children with uncomplicated malaria in coastal Ghana
Source: Malar J. 2016 Dec 9;15:592. doi: 10.1186/s12936-016-1640-8 (PMC5148883; doi:10.1186/s12936-016-1640-8)
Supplement: Supplementary file 1 — Additional file 1. Primer list [file 12936_2016_1640_MOESM1_ESM.docx]

**Additional file 1**

*msp1* *msp2* genotyping primers, *Pfs25* RT-PCR, *Pfs4845*, human blood group O primers and G6PD primers

| **Gene/primer name** | | | **Primer sequence** | **Annealing temp. (^0^C)** | |
| --- | --- | --- | --- | --- | --- |
| ***msp1*** | | |  |  | |
| outer M1- OF | | | CTAGAAGCTTTAGAAGATGCAGTATTG | 54 | |
| M1- OR | | | CTTAAATAGTATTCTAATTCAAGTGGATCA |  | |
| K1 M1- KF | | | AAATGAAGAAGAAATTACTACAAAAGGTGC | 59 | |
| M1- KR | | | GCTTGCATCAGCTGGAGGGCTTGCACCAGA |  | |
| MAD 20 M1- MF | | | AAATGAAGGAACAAGTGGAACAGCTGTTAC | 59 | |
| M1- MR | | | ATCTGAAGGATTTGTACGTCTTGAATTACC |  | |
| RO33 M1- RF | | | TAAAGGATGGAGCAAATACTCAAGTTGTTG | 59 | |
| RO33- R2 | | | CAAGTAATTTTGAACTCATGTTTTAAATCAGCGTA |  | |
| ***msp2*** | | |  |  | |
| Outer M2- OF | | | ATGAAGGTAATTAAAACATTGTCTATTATA | 54 | |
| M2- OR | | | CTTTGTTACCATCGGTACATTCTT |  | |
| 3D7/FC27 S1fw | | | GCTTATAATATGAGTATAAGGAGAA | 50 | |
| FC27 M5rev | | | GCATTGCCAGAACTTGAA |  | |
| 3D7 N5rev | | | CTGAAGAGGTACTGGTAGA |  | |
| **RT- PCR** | | |  |  | |
| *Pfs25* Pfs25F | | | TCTTTTCCTTTTCATTCAACTTAGCA | 60 | |
| Pfs25R | | | CCACTCATCTGAATTAAAAATCCTCTT |  | |
| *Pfs48/45* SF1 | | | TCGGAATTAGGTTTAATTGAATATG | 60 | |
| OR2 | | | GTCGTTTTTGGTATACTTCCAACT |  | |
| **Blood grouping** | | |  |  | |
| BgOF | | | CACCGTGGAAGGATGTCCTC | 58 | |
| BgOR | | | \| AATGTCCACAGTCACTCGCC \| \| --- \| |  | |
| **G6PD** |  | | |  | |
| 376F | CCCAGGCCACCCCAGAGGAGA | | | 61 | |
| 376R | \| CGGCCCCGGACACGCTCATAG \| \| --- \| | | |  | |
| 202F | CCACCACTGCCCCTGTGACCT | | | 65 | |
| 202R | \| GGCCCTGACACCACCCACCTT \| \| --- \| | | |  | |
| 680F | ACATGTGGCCCCTGCACCAC | | | 69 | |
| 680R | \| GTGACTGGCTCTGCCACCCTG \| \| --- \| | | |  | |
| 968F | TCCCTGCACCCCAACTCAAC | | | 65 | |
| 968R | \| CCAGTTCTGCCTTGCTGGGC \| \| --- \| | | |  | |
|  |  | | |  |  |
|  |  | | |  |  |
