# Supplementary material for: Plasmodium falciparum genotype and gametocyte prevalence in children with uncomplicated malaria in coastal Ghana
Source: Malar J. 2016 Dec 9;15:592. doi: 10.1186/s12936-016-1640-8 (PMC5148883; doi:10.1186/s12936-016-1640-8)
Supplement: Supplementary file 2 — Additional file 2. Validation of the Pfs25 mRNA primer set [file 12936_2016_1640_MOESM2_ESM.docx]

PF3D7_1031000 (PF10_0303) Product: 25 kDa ookinete surface antigen precursor (Pfs25)


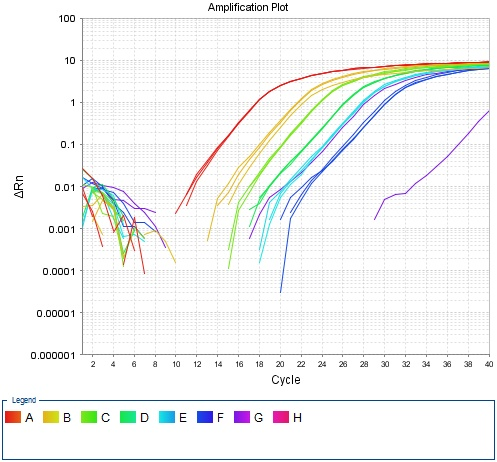

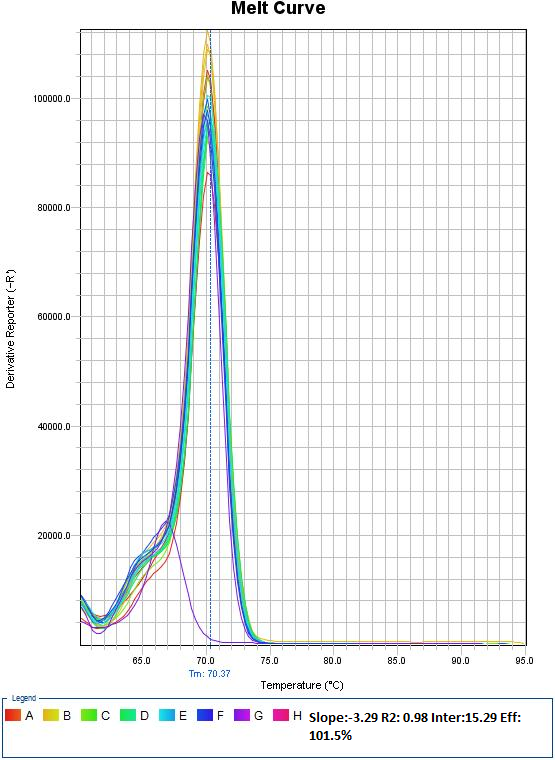

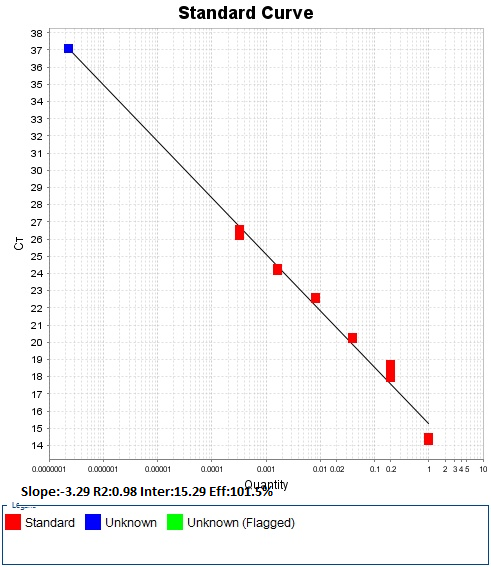


Slope=-3.29

Y-int= 15.29

R^2^ = 0.98

Eff. %= 101.5

Starting concentration 1:20 dilution 1:5 Primer concentration 300 nM

Fast Sybr Run Method:
Holding: 95° for 20 sec Denature: 95 ° for 3 sec Anneal/Extend: 60° for 30 sec *40 cycles

Gametocytes cDNA Starting concentration 1:20 with two fold serial dilution (1:2)
