# Supplementary material for: Plasmodium falciparum genotype and gametocyte prevalence in children with uncomplicated malaria in coastal Ghana
Source: Malar J. 2016 Dec 9;15:592. doi: 10.1186/s12936-016-1640-8 (PMC5148883; doi:10.1186/s12936-016-1640-8)
Supplement: Supplementary file 3 — Additional file 3. Statistical analysis [file 12936_2016_1640_MOESM3_ESM.docx]

A. Relationship between age and multiplicity of infection (MOI) and parasite density (PD) for all the children

|  | MSP1 MOI | | | |  | MSP2 MOI | | |  | PD | | |
| --- | --- | --- | --- | --- | --- | --- | --- | --- | --- | --- | --- | --- |
| Age stratification* | Count | | Mean (SEM) | p value |  | Count | Mean (SEM) | p value |  | Count | Mean (SEM) | p value |
| 4-60 months | 52 | | 2.019 (0.1273) | 0.2149 |  | 52 | 2.385 (0.1906) | 0.1063 |  | 40 | 87550 (24619) | 0.9305 |
| 72-154 months | 36 | | 2.278 (0.1624) |  |  | 42 | 1.929 (0.1613) |  |  | 32 | 55265 (13585) |  |
| B. Relationship between age and PD and CT values obtained for Pfs25 reverse transcriptase PCR on D0 and D7 for the subset of 35 children | | | | | | | | | | | | |
|  | | D0 CT | | |  | D7 CT | | |  |  |  |  |
| Age stratification# | | Count | Mean (SEM) | p value |  | Count | Mean (SEM) | p value |  |  |  |  |
| 8-60 months | | 19 | 31.85 (1.477) | 0.6169 |  | 19 | 35.76 (1.222) | **0.0292** |  |  |  |  |
| 72 - 144 months | | 16 | 31.06 (1.028) |  |  | 16 | 32.43 (0.901) |  |  |  |  |  |
| C. Relationship between PD and CT values obtained for Pfs25 reverse transcriptase PCR for the subset of 35 children | | | | | | | | | | | | |
|  | D0 CT | | | |  | D7 CT | | |  |  |  |  |
| PD stratification | Count | | Mean (SEM) | p value |  | Count | Mean (SEM) | p value |  |  |  |  |
| <34052 | 23 | | 31.76 (1.05) | 0.6739 |  | 23 | 33.73 (0.9108) | 0.7229 |  |  |  |  |
| >34052 | 12 | | 30.95 (1.578) |  |  | 12 | 34.38 (1.532) |  |  |  |  |  |

D. Relationship between MOI and CT values obtained for Pfs25 reverse transcriptase PCR on day 0 and day 7 for the subset of 35 children

|  | Best-fit values ± SE | DO CT | D7 CT |
| --- | --- | --- | --- |
| MSP1 MOI | slope | -0.06991 ± 1.098 | 0.8938 ± 0.9817 |
|  | p value | 0.9497 | 0.3701 |
| MSP2 MOI | slope | -0.2819 ± 0.7802 | -0.3559 ± 0.6876 |
|  | p value | 0.7203 | 0.6083 |

* Total children, # Children used in submicroscopic gametocyte analysis, p value predicted using Mann-Whitney test for A, B and C and Linear regression for D. PD, parasite density observed by microscopy per microliter of blood; MOI, multiplicity of infection; D0 CT, value of the CT (cycle threshold) obtained during the Pfs25 reverse transcriptase real time PCR on day 0 (increase in CT value suggests a negative association with submicroscopic gametocytes); D7 CT, value of the CT obtained during the Pfs25 reverse transcriptase real time PCR on day 7; MOI-MSP1 is the multiplicity of infection based on *msp*1 genotyping; MOI-MSP2 is the multiplicity of infection based on *msp*2 genotyping.

Variables are reported as means, with the standard error of the mean indicated in parenthesis. The count represents the total number of children in each category. Children were stratified into two age groups, those 5 years and below (4 – 60 months) and those 6 years and above (72 – 154 months). In table C, parasite density (PD) was stratified by the geometric mean (34052/μl) of parasites contained in the 35 children on day 0.
