# Supplementary material for: Plasmodium falciparum genotype and gametocyte prevalence in children with uncomplicated malaria in coastal Ghana
Source: Malar J. 2016 Dec 9;15:592. doi: 10.1186/s12936-016-1640-8 (PMC5148883; doi:10.1186/s12936-016-1640-8)
Supplement: Supplementary file 4 — Additional file 4. Features of samples analyzed for submicroscopic gametocytes [file 12936_2016_1640_MOESM4_ESM.docx]

**Additional file 4:** Parasite CT values by RT-PCR amplification *Pfs2*5 gene, Age, *msp* MOI and G6PD genotype of the 35 subset samples.

| **sample ID** | **sex** | **Age (Month)** | **D0 parasite density/uL of blood** | ***msp* 1 MOI** | ***msp* 2 MOI** | **AVG. Pfs25 CT D0** | **AVG. Pfs25**  **CT D7** | **G6DP** |
| --- | --- | --- | --- | --- | --- | --- | --- | --- |
| EW 049 | M | 144 | 81120 | 2 | 1 | 30.5 | 34 | B |
| EW 052 | M | 60 | 7920 | 1 | 2 | 30.8 | 34.5 | A |
| EW 045 | M | 21 | 9480 | 1 | 1 | 30.8 | 32.6 | B |
| EW 005 | F | 36 | 30160 | 1 | 1 | 25.7 | 32.5 | BB |
| EW 011 | M | 72 | 3440 | 3 | 2 | 24.5 | 27 | B |
| EW 048 | F | 108 | 6480 | 3 | 2 | 34 | 36 | BB |
| ELM 3244 | M | 108 | 295440 | 3 | 2 | 23 | 33 | B |
| EW 050 | M | 84 | 166080 | 3 | 1 | 33 | 26.7 | A- |
| ELM 3606 | F | 108 | 0 | 2 | 3 | 29 | 36 | AA |
| EW 035 | F | 19 | 0 | 1 | 1 | 32 | 28 | AA- |
| ELM 3226 | M |  | 0 | 2 | 6 | 24 | 37 | A |
| EW 060 | F | 20 | 0 | 2 | 3 | 35 | 32 | AB |
| EW 057 | F | 96 | 0 | 3 | 3 | 30 | 32.7 | AA |
| ELM 3223 |  |  | 0 |  | 2 | 38 | 38 | _ |
| EW 046 | F | 15 | 10240 | 1 | 1 | 27.8 | 31 | BB |
| GC 14320 | M | 26 | 0 | 3 | 2 | 38 | 32 | B |
| GC 13769 | M | 72 | 0 |  | 1 | 32 | 40 | B |
| EW 065 | F | 96 | 158480 | 3 | 3 | 29 | 28.8 | AB |
| EW 062 | M | 48 | 172720 |  | 2 | 28.9 | 31 | B |
| GC 13419 | M | 108 | 6560 | 1 | 1 | 40 | 40 | B |
| GC 13116 | M | 72 | 0 | 2 | 2 | 40 | 40 | A- |
| GC 13228 | M | 108 | 55920 |  | 4 | 40 | 40 | B |
| ELM 3471^P,M^ | F | 72 | 64080 | 2 | 1 | 23 | 40 | BB |
| EW 064 | F | 8 | 122560 | 1 | 2 | 28 | 29 | BB |
| EW 028 | F | 48 | 14480 | 2 | 3 | 40 | 30.5 | AB |
| GC 13360 | F | 84 | 192640 | 2 | 2 | 35 | 40 | BB |
| GC 13856^P^ | F | 48 | 648080 | 3 | 2 | 30 | 40 | AA |
| GC 14325 | M | 120 | 0 | 3 | 2 | 28.7 | 38 | B |
| GC 13516 | M | 60 | 3680 | 2 |  | 30.7 | 40 | B |
| EW 018 | M | 12 | 54720 | 1 | 5 | 31 | 30 | B |
| EW 007 | F | 48 | 0 | 3 | 2 | 29 | 31 | AA- |
| EW 034 | F | 13 | 21920 | 1 | 3 | 36 | 27 | AB |
| EW 022 | M | 24 | 0 | 3 | 3 | 29 | 33 | A |
| EW 032^G^ | F | 13 | 2240 | 1 | 3 | 25.5 | 27 | BB |
| GC 13589 | F | 108 | 59840 | 3 | 1 | 40 | 40 | AA- |
| **control gcyte** |  |  |  |  |  | **15.7** | **15.7** |  |
| **control NTC** |  |  |  |  |  | 40 | 38 |  |

* samples have been arranged in order of increasing Pfs48/45 antibody titres; ^P^ samples positive for parasites by PCR on day 7; ^M^ samples positive for asexual parasites by microscopy on day 7; ^G^ sample positive for gametocytes on day 0 and day 7.
